# Hanle Detection for Optical Clocks

**DOI:** 10.1155/2015/614737

**Published:** 2015-02-03

**Authors:** Xiaogang Zhang, Shengnan Zhang, Duo Pan, Peipei Chen, Xiaobo Xue, Wei Zhuang, Jingbiao Chen

**Affiliations:** State Key Laboratory of Advanced Optical Communication Systems and Networks, Institute of Quantum Electronics, School of Electronics Engineering & Computer Science, Peking University, Beijing 100871, China

## Abstract

Considering the strong inhomogeneous spatial polarization and intensity distribution of spontaneous decay fluorescence due to the Hanle effect, we propose and demonstrate a universe Hanle detection configuration of electron-shelving method for optical clocks. Experimental results from Ca atomic beam optical frequency standard with electron-shelving method show that a designed Hanle detection geometry with optimized magnetic field direction, detection laser beam propagation and polarization direction, and detector position can improve the fluorescence collection rate by more than one order of magnitude comparing with that of inefficient geometry. With the fixed 423 nm fluorescence, the improved 657 nm optical frequency standard signal intensity is presented. The potential application of the Hanle detection geometry designed for facilitating the fluorescence collection for optical lattice clock with a limited solid angle of the fluorescence collection has been discussed. The Hanle detection geometry is also effective for ion detection in ion optical clock and quantum information experiments. Besides, a cylinder fluorescence collection structure is designed to increase the solid angle of the fluorescence collection in Ca atomic beam optical frequency standard.

## Introduction

The Hanle effect, dating back to 1923 [1], has contributed much in the development of atomic physics and, more generally, of quantum mechanics. Hanle had first correctly elaborated the effect named after him in 1924 [2]. The Hanle effect is mainly known as a depolarization of resonance fluorescence as the Zeeman states of the excited atomic energy levels become degenerate when an applied magnetic field is swept through zero. The Hanle effect has been used to precisely measure the lifetimes of excited atomic states [3–5]. Its extension to nonzero magnetic fields, the level-crossing technique, made possible precise sub-Doppler measurements of fine and hyperfine structure separation [6, 7]. It plays an irreplaceable role in modern atomic spectroscopy [7–13]. Pierre Cérez et al. found that the optical pumping efficiency as well as the fluorescence yield is sensitive to the Hanle effect in the Cesium beam frequency standard [14–16].

In our proposed Ca atomic beam optical frequency standard [17] with the electron-shelving method [18], we use 423 nm ^1^ *S*_0_ → ^1^ *P*_1_ transition signal to obtain the 657 nm ^1^ *S*_0_ → ^3^ *P*_1_ clock transition signal, which is the frequency standard. Based on the same ground state which 423 nm transition and 657 nm clock transition have and the stronger transition of the 423 nm transition than 657 nm clock transition, the enhanced fluorescence signal of the 423 nm transition is observed when atoms are in the ground state, while no fluorescence occurs when atoms are “shelved” in the 657 nm clock transition excited state. This method extracts the 657 nm frequency standard signal from the 423 nm transition signal and improve the signal to noise ratio of the 657 nm frequency standard signal. The electron-shelving method has been the most common detection method in current neutral and ion optical clocks [19–23].

Even the important role of the Hanle effect played in microwave atomic clock has been investigated clearly [14, 15]; however, the strong influence of the Hanle effect on the fluorescence collection rate of the electron-shelving method has never been discussed in Ca atomic beam optical frequency standard [17, 24], or the other optical lattice clocks [19–22]. In this paper, it is demonstrated that the Hanle effect strongly affects the detection efficiency of electron-shelving method in a compact Ca atomic beam optical frequency standard. We propose and demonstrate a universe Hanle detection geometry of the electron-shelving method for optical clocks. The Hanle detection geometry with optimized magnetic field direction, detection laser beam propagation and polarization direction, and detector position can improve the detected fluorescence intensity by more than one order of magnitude comparing with that of inefficient geometry in optical clocks. The improved 657 nm optical frequency standard signal is presented with the Hanle detection geometry. Besides, we design a fluorescence collection structure to improve the solid angle of the fluorescence collection in Ca atomic beam optical frequency standard. Both collection approaches will improve the signal to noise ratio of the optical clock signal.

Nowadays, the optical lattice clock has achieved 10^−18^ stability and uncertainty [19, 20], which is the best performance of optical clocks. With the Hanle detection geometry, a high signal to noise ratio of the frequency standard signal can be achieved with the enhanced fluorescence intensity. This will be very helpful to further optimize the performance of optical clocks [19–22]. The Hanle detection geometry is also effective for ion detection in ion optical clock and quantum information experiments [23, 25, 26].

## 2. Hanle Detection in Ca Atomic Beam Optical Frequency Standard

**Figure 1:**
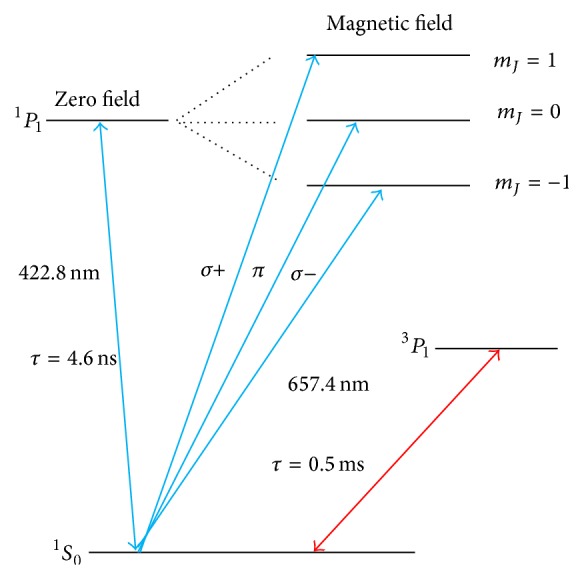
Relevant Ca energy levels.

**Figure 2:**
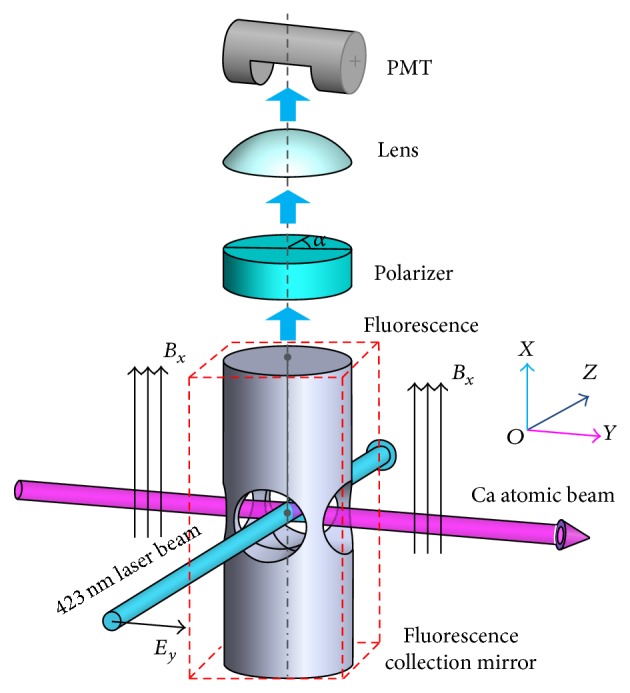
The Hanle detection configuration. The cylinder fluorescence collection structure with two through-holes in the interaction area in the dashed box is added for increasing the solid angle of the fluorescence collection. Without the cylinder fluorescence collection structure, the Hanle effect is measured.

In Ca atomic beam optical frequency standard [17], we collect the 423 nm transition fluorescence signal to steer the 657 nm frequency standard signal. The relevant energy levels are shown in Figure 1. With the presence of the magnetic field, the excited state ^1^ *P*_1_ is split into three Zeeman sublevels. The whole 423 nm fluorescence collection system is shown in Figure 2. In Figure 2, the orientation of the homogeneous magnetic field is in the $\hat{x}$-direction. The Ca atomic beam is along the $\hat{y}$-direction. The incident laser travels along the $\hat{z}$-direction and a linear polarization orientation can be changed in the $\hat{x}\text{-}\hat{y}$ plane. After the interaction between Ca atomic beam and 423 nm laser, the photomultiplier (PMT) collects the fluorescence radiation along the $\hat{x}$-direction. Besides, the polarizer in Figure 2 is only used for demonstrating the Hanle effect. When we detect all fluorescence signal for optical frequency standard operating, the polarizer is removed.

**Figure 3:**
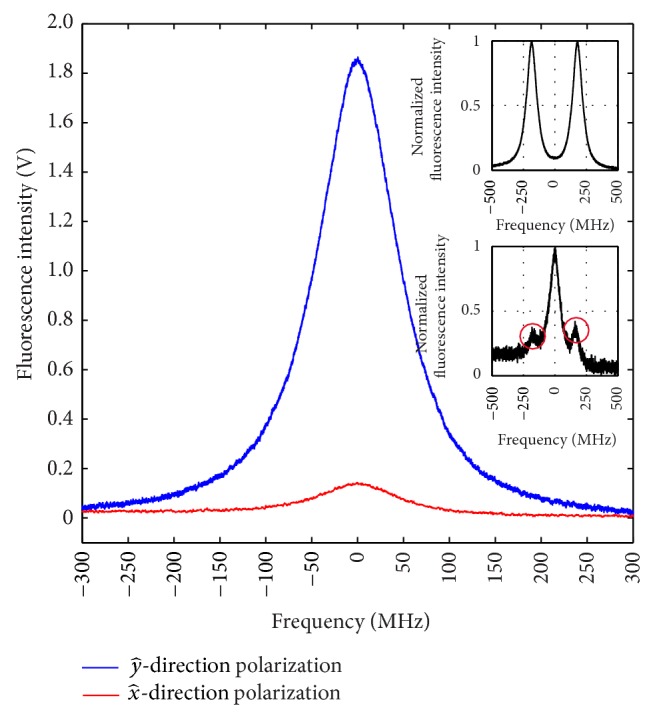
The detected fluorescence signal in different polarization orientation of the 423 nm detection laser with zero magnetic field. The upper blue fluorescence signal is collected when the polarization orientation of the 423 nm laser is in the $\hat{y}$-direction; the lower red fluorescence signal is collected when the polarization orientation of the 423 nm laser is in the $\hat{x}$-direction; The upper inset is the detected fluorescence signal as the polarization orientation of the 423 nm laser is in the $\hat{y}$-direction while the magnetic field is 121.6 G. The lower inset is the detected fluorescence signal as the polarization orientation of the 423 nm laser is in the $\hat{x}$-direction while the magnetic field is 121.6 G.

In our preliminary fluorescence collection system, the cylinder fluorescence collection structure with two through-holes in the dashed box as shown in Figure 2 is absent. Without the cylinder fluorescence collection structure, the detected fluorescence intensity of PMT changed as the linear polarization orientation of 423 nm laser changed without the polarizer. The maximum fluorescence intensity occurred with the $\hat{y}$-direction polarization of the detection laser while the minimum intensity occurred with the $\hat{x}$-direction polarization of the detection laser. The detected fluorescence intensity of PMT changing with 423 nm laser polarization near zero static magnetic field is shown in Figure 3. In Figure 3, the detected fluorescence intensity with the $\hat{y}$-direction polarization of the detection laser is 15.8 times larger than the detected fluorescence intensity with the $\hat{x}$-direction polarization of the detection laser. The frequency range is calibrated by the Ca atom isotope. The 423 nm transition frequency of ^40^Ca and ^44^Ca isotope is apart from 773.8 MHz [27].

With fixed magnetic field orientation and fixed laser propagation direction, the detected fluorescence intensity under zero magnetic field changes with the laser polarization orientation. In the classical theory, we could take Ca atoms in the laser field as a classical dipole oscillator that is set in motion parallel to the direction of the polarization of the exciting laser. The radiation emitted by the oscillator will be polarized in the same direction as that of the incident laser, but it cannot radiate in the direction of its vibration. In Figure 3, the results show little fluorescence intensity can be collected when the polarization orientation of the detection laser is in the $\hat{x}$-direction because the polarization orientation of the detection laser is not exactly in the $\hat{x}$-direction and there is residual earth magnetic field. Once there is an external magnetic field, the oscillator processes around the magnetic field and the polarization of the fluorescence will change. The intensity of the fluorescence can be expressed as [6]

(1) IB=C∫0∞I(B,t)dt=CI02∫0∞exp⁡−Γt1−cos⁡2wLt−αdt=CI021Γ−cos⁡2αΓΓ2+4wL2−sin2α2wLΓ2+4wL2,

where *I*_0_ is the initial intensity and *C* is the constant for the fluorescence collection. Γ is the spontaneous radiation linewidth of the excited state. For the Ca 423 nm transition, Γ = 2*π* × 34.6 MHz. *w*_*L*_ = *g*_*J*_(*μ*_*B*_/*ħ*)*B* is the Larmor frequency and *μ*_*B*_ = *eħ*/2*m*_*e*_ is the Bohr magneton. For observing the Hanle effect, we added a polarizer in front of the PMT. Through a polarizer, *α* is the angle between the transmission axis of the polarizer and the polarization orientation of the fluorescence.

**Figure 4:**
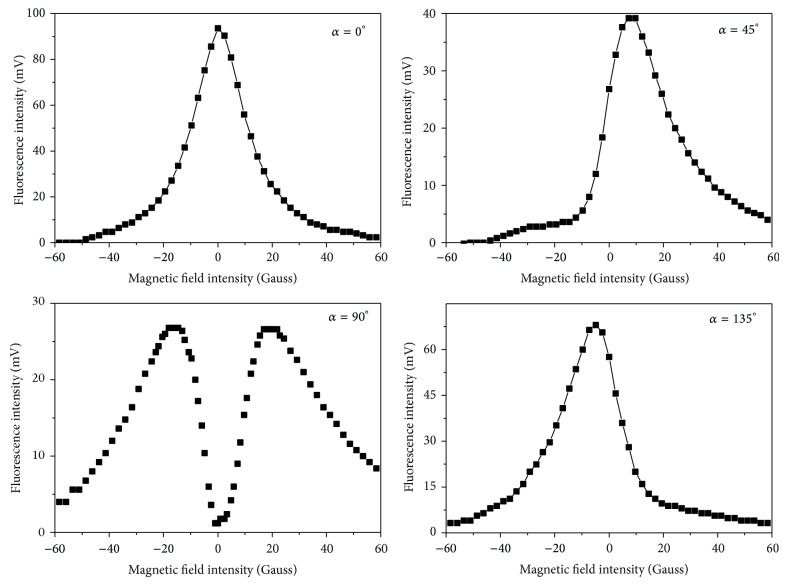
The Hanle effect signal in different *α*. The Lorentzian shape is observed for *α* = 0° and 90°. The incomplete dispersion shape is observed for *α* = 45° and 135°.

Thus, the shape of the Hanle effect signal with the magnetic field being swept through zero depends on the orientation of the polarizer. As shown in Figure 4, the Hanle effect signals have a Lorentzian shape for *α* = 0° and 90° and a dispersion shape for *α* = 45° and 135° while the magnetic field is swept through zero. The reason for the incomplete dispersion shape is that the increasing Zeeman splitting with the increasing magnetic field decreases the signal intensity within the finite Doppler broadened spectra.

**Figure 5:**
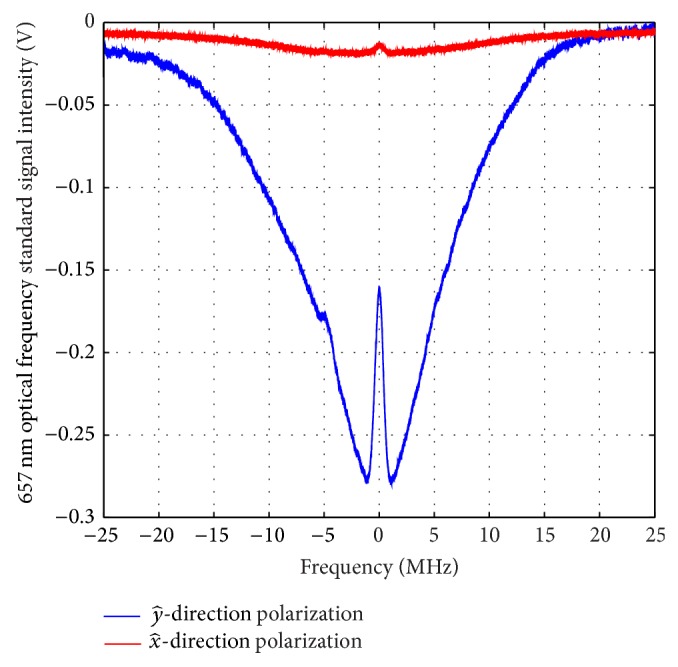
The detected 657 nm optical frequency standard signal by using 423 nm electron-shelving method in Ca atomic beam optical frequency standard with and without the Hanle detection geometry. The 657 nm light is from the semiconductor laser.

While the 423 nm laser is locked to the 423 nm atomic transition line, we detect the 657 nm saturation spectroscopy signal with electron-shelving method. A similar system setup has been shown in [17]. The polarization of the 657 nm laser light is in $\hat{x}$-direction, which is parallel to the magnetic field. The Δ*m* = 0 transition is immune to the change of the magnetic field. In Figure 5, the detected 657 nm saturation spectroscopy intensity with the $\hat{y}$-direction polarization of the 423 nm detection laser is 20.4 times larger than 657 nm saturation spectroscopy intensity with the $\hat{x}$-direction polarization of the 423 nm detection laser, which will bring one order of magnitude optimization in the performance of Ca atomic beam optical frequency standard. With the Hanle detection geometry based on the electron-shelving method, the fluorescence collection rate is much more improved than the standard electron-shelving method.

## 3. The Cylinder Fluorescence Collection Structure

Based on the Hanle effect in Ca atomic beam optical frequency standard, the most effective fluorescence collection geometry as shown in Figure 2 is that the 423 nm detection laser should be polarized in $\hat{y}$-direction while the magnetic field and the PMT is in the $\hat{x}$-direction. An optimized polarization orientation of the detection laser should be chosen for a fixed magnetic field direction and detector position, or an optimized detector position should be chosen for a fixed magnetic field direction and polarization orientation of the detection laser in a settled optical clock. The basic principle of the Hanle detection geometry is to set the PMT at the plane which is perpendicular to the polarization direction of the detection laser which is parallel to the magnetic field direction, or at the position along the magnetic field direction while the magnetic field direction is perpendicular to the polarization direction of the detection laser. Besides, we design a fluorescence collection structure for increasing the solid angle of the fluorescence collection in the compact Ca atomic beam optical frequency standard.

In Figure 2, the cylinder fluorescence collection structure with two through-holes in the dashed box is added. In the bottom of the cylinder, there is a 95% reflection concave mirror. Also, in the top of the cylinder, there is a 95% reflection concave mirror with a hole for the output of the fluorescence to PMT. Because the laser beam and atomic beam are in the $\hat{x}\text{-}\hat{y}$ plane, we can only collect the fluorescence in the $\hat{z}$-direction, that is, on the top of cylinder fluorescence collection structure. The two reflection mirrors increase the solid angle of the fluorescence collection and average out spatial fluorescence distribution of the Hanle effect. Comparing with Figure 3, the results show that the detected fluorescence intensity with the $\hat{y}$-direction polarization of the detection laser is only 1.3 times larger than the detected fluorescence intensity with the $\hat{x}$-direction polarization of the detection laser. The influence of the Hanle effect is averaged. This structure is only to increase the solid angle of the fluorescence collection to collect more fluorescence signal. The Hanle detection is still working in this structure.

## 4. Potential Application of the Hanle Detection for the Optical Lattice Clock

**Figure 6:**
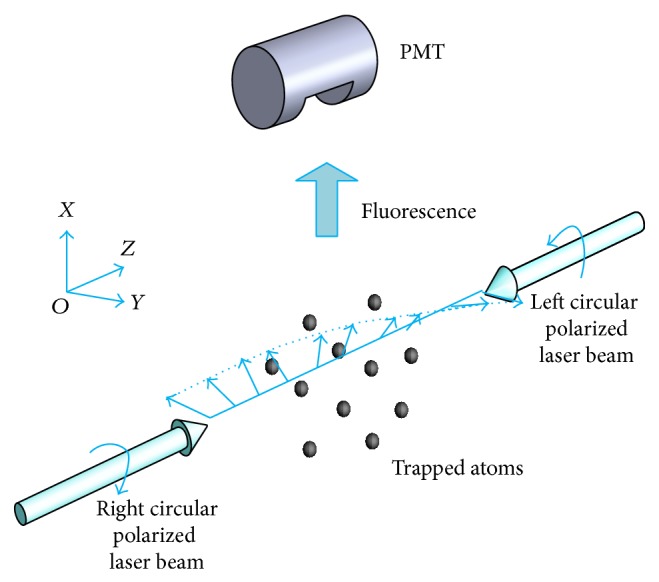
A normal fluorescence collection configuration of electron-shelving method in Sr optical lattice clock.

Currently, the best performance of optical lattice clocks has reached 10^−18^ stability and uncertainty [19, 20]. In order to further optimize the performance of the optical lattice clock [19–22], the improved fluorescence collection rate will be helpful. A normal fluorescence collection configuration of electron-shelving method in optical lattice clock [21] is shown in Figure 6, where two counterpropagation detection lasers are circular polarization and in opposite polarization orientation. In this case, the detection laser can be taken as a linear polarization laser and rotates around the propagation of the laser. The maximum fluorescence intensity is in the propagation direction of the detection laser. When the PMT is set to the position in Figure 6, only half of the fluorescence intensity is detected. If we use an independent linearly polarization detection laser in optical lattice clocks [19–22], the most efficient Hanle detection geometry is to set the PMT at the plane which is perpendicular to the polarization direction of the detection laser which is parallel to the magnetic field direction, or at the position along the magnetic field direction while the magnetic field direction is perpendicular to the polarization direction of the detection laser. The second choice is the previous configuration with the PMT in the propagation direction of the detection laser. Otherwise, the fluorescence collection rate will decrease by more than one order of magnitude with the inefficient geometry. Our experimental results demonstrate the Hanle detection geometry can improve the fluorescence collection rate for the optical lattice clock and further optimize the performance of the optical lattice clock by increasing the detected signal intensity to its maximum value.

## 5. Conclusion

In conclusion, as in microwave atomic clock [14, 15], Hanle effect also plays an important role in optical clock. Due to the Hanle effect, the fluorescence collection rate which changes 15.8 times is observed in the compact Ca atomic beam optical frequency standard. We thus propose the Hanle detection geometry of the electron-shelving method for optical clocks with limited solid angle of the fluorescence collection. The principle of the Hanle detection is to set the PMT at the plane which is perpendicular to the polarization direction of the detection laser which is parallel to the magnetic field direction, or at the position along the magnetic field direction while the magnetic field direction is perpendicular to the polarization direction of the detection laser, which is the most efficient Hanle detection geometry. Also, one can use the opposite circular polarization detection laser in counterpropagation direction with the PMT in the propagation direction of the detection laser. With the Hanle detection geometry, the 657 nm optical frequency standard signal intensity is improved 20 times than the normal electron-shelving method, which will optimize the performance of the Ca atomic beam optical frequency standard. It is also effective for the optical lattice clock, ion clock, and quantum information experiments [19–23, 25, 26]. The Hanle detection geometry will be helpful to further improve the fluorescence collection rate of optical clocks [19–22]. Besides, we design a fluorescence collection structure to improve the solid angle of the fluorescence collection in the compact Ca atomic beam optical frequency standard for collecting more fluorescence.
